# A methodology for experimental evaluation of signal detection methods in spectrum sensing

**DOI:** 10.1371/journal.pone.0199550

**Published:** 2018-06-22

**Authors:** Tomaž Šolc, Mihael Mohorčič, Carolina Fortuna

**Affiliations:** 1 Department of Communication Systems, Jožef Stefan Institute, Jamova cesta 39, 1000 Ljubljana, Slovenia; 2 Jožef Stefan International Postgraduate School, Jamova cesta 39, 1000 Ljubljana, Slovenia; Beijing University of Posts and Telecommunications, CHINA

## Abstract

Lack of unallocated spectrum and increasing demand for bandwidth in wireless networks is forcing new devices and technologies to share frequency bands. Spectrum sensing is a key enabler for frequency sharing and there is a large body of existing work on signal detection methods. However a unified methodology that would be suitable for objective comparison of detection methods based on experimental evaluations is missing. In this paper we propose such a methodology comprised of seven steps that can be applied to evaluate methods in simulation or practical experiments. Using the proposed methodology, we perform the most comprehensive experimental evaluation of signal detection methods to date: we compare energy detection, covariance-based and eigenvalue-based detection and cyclostationary detection. We measure minimal detectable signal power, sensitivity to noise power changes and computational complexity using an experimental setup that covers typical capabilities from low-cost embedded to high-end software defined radio devices. Presented results validate our premise that a unified methodology is valuable in obtaining reliable and reproducible comparisons of signal detection methods.

## 1 Introduction

In recent years, we witnessed a drastic growth in the demand for bandwidth in wireless communications, mostly in consumer devices. Due to the lack of unallocated spectrum, frequency bands are increasingly shared between different services [1]. Often frequencies are shared with legacy devices that have been designed under the assumption of exclusive frequency use. New devices and technologies hence face an interference avoidance problem. Spectrum sensing is a promising approach to solving this problem. It allows a device to detect the presence of other users and adapt its use of spectrum accordingly.

Spectrum sensing technology has seen mixed success in the past years. On one hand, it has been successfully introduced in a number of widely deployed devices: dynamic frequency selection (DFS) feature of the IEEE 802.11h standard [2] allows Radio LANs (RLANs) to coexist with radars in the 5 GHz UNII frequency band through spectrum sensing. Many commercial IEEE 802.11 access points now include automatic channel selection [3] based on proprietary spectrum sensing algorithms. On the other hand, spectrum sensing in TV white spaces (TVWS) has seen only limited success [4] despite the amount of attention received in the United States and, more recently, in the United Kingdom [5]. Today, commercial TVWS devices almost exclusively depend on geolocation databases to address the interference problem. The IEEE 802.22 standard that includes spectrum sensing capabilities [6], has also not seen wide-spread adoption.

Despite the recent failure in being widely adopted in TVWS, spectrum sensing is still interesting for future technologies. It is more economic to implement than geolocation database access particularly for small and battery powered devices [7]. Such devices are key to the so-called Internet of Things. Geolocation databases can also be problematic where infrastructure access and reliability is an issue, for example in Vehicle to Vehicle (V2V) communication [8]. Spectrum sensing has also been identified as one of enabling technologies for efficient spectrum sharing in 5G mobile systems [9], including machine type communication (MTC).

### 1.1 Motivation

The motivation for our work is two-fold.

Firstly, in spite of a large body of studies on spectrum sensing, several representatives of the research community agree there is no standard way of designing and comparing signal detection methods. A variety of different evaluation approaches are being used in the literature. The results from different studies are thus hard, if not impossible to objectively compare. This situation is calling for a *common methodology* for designing and evaluating signal detection methods, similarly to what we can find in many other research areas related to wireless networks.

Secondly, we noticed that many analyses based only on theory and simulations [10–13] fail to account for the effects of practical receiver designs on signal detection performance. By introducing a methodology that also covers practical validation, we hope to encourage experimentation in this field that will eventually result in a more solid understanding of signal detection methods.

### 1.2 Contributions

The *main* contribution and a novelty of this paper is a *unified methodology*, which is based on commonly used yet thus far not harmonised and generalised steps. This methodology can be used to resolve the existing tussles around various signal detection methods by means of uniform objective quantitative evaluation and comparison. We focus solely on blind signal detection, which we define as a form of signal reception where we are only interested in the fact that a transmission exists and not in the information it carries. The proposed methodology is comprised of seven steps and can be applied to evaluate methods in simulation or practical experiments. Some steps can be seen as examples of good practice leading to reproducibility of results [14]. We show that these steps are even more important when it comes to fair comparison of methods that differ significantly in their implementation.

Another *major* contribution of this work is a *comprehensive experimental evaluation* of analog and digital energy detection, covariance-based and eigenvalue-based detection and cyclostationary detection. We measure minimal detectable signal power for signals with two typical analog and digital modulations, sensitivity of detection methods to noise power changes and their computational complexity. We use an experimental setup that covers typical capabilities from embedded to high-end software defined radio devices. We also experimentally evaluate the effect of filter compensation on covariance-based detection. In order to support reproducibility and cross-comparison of existing and new spectrum sensing methods, we openly publish the source code of our implementations of signal detectors and signal models.

The rest of the paper is organized as follows. Section 2 discusses how existing literature on methodology and spectrum sensing relates to our work. Section 3 describes the proposed methodology with the subsequent sections describing individual steps in our specific evaluation. Section 4 lists the selected detection methods using a common form. Section 5 describes the signal and noise model waveforms. Our implementation of detection methods and waveforms is described in Section 6. The experimental setup is outlined in Section 7. The evaluation procedures and results are presented in Sections 8 and 9 respectively. Finally, Section 10 concludes the paper with a summary of our main findings.

## 2 Related work

In this section, we provide an overview of previously published work related to methodologies and spectrum sensing. Existing literature on spectrum sensing is very rich, hence we limit ourselves only to publications that are most relevant to our work. For a comprehensive survey of the broader topic of spectrum sensing we direct readers to [15]. An excellent review of the mathematical background for many detection methods is available in [16].

### 2.1 On methodology

In the wireless network literature authors have proposed various methodologies for developing new models and technologies. The authors of [17] propose a design methodology that enables developing more efficient and dependable receivers using cross-layer design. In [18], the author proposes a design methodology for highly integrated low-power receivers for wireless communications, while in [19] the authors propose a design methodology for using TCP over wireless links that have a link level error control mechanism.

The authors of [20] propose a methodology for optimizing wireless sensor networks dynamically using Markov Decision Processes. In [21] a multi-purpose testing methodology for cellular network components is introduced and demonstrated by measuring the data rate under different experimental conditions. In [22] authors propose a methodology for spectrum occupancy surveys, mostly focusing on energy detection, with the aim of producing directly comparable quantitative results.

All these works have a different scope than the one proposed in this paper. Our work is the first of its kind by proposing a methodology for uniform, scientifically objective evaluation of the large number of existing signal detection methods that promotes reproducible repetitions and re-evaluations, thus also representing a novelty in the field.

The closest related work to ours is [10], where several blind detection methods are reduced to a common analytic form to aid their comparison. The work in [10] represents only the very first steps required to objectively study the performance of existing methods. While their work identifies detection methods, presents a common formalization and signal models, it does not address the implementation, simulation and/or experimental and evaluation procedures. Compared to [10], our work not only proposes a generic methodology covering all necessary steps but it also demonstrates the validity of the methodology on the largest number of signal detection methods evaluated against each other thus far.

### 2.2 On spectrum sensing

Spectrum sensing has several definitions, perhaps the most representative being the *“ability of a device to listen for other nearby spectrum users to determine whether it is possible to transmit”* [23] and, more broadly, as *“a task of obtaining awareness about the spectrum usage and operational electromagnetic environment”* [24].

Various aspects of spectrum sensing have been widely covered in recent literature, both in the form of surveys, papers focusing on individual methods and papers focusing on TVWS use. A vast majority of existing work, however, is based on theoretical models and simulations.

In [15], a comprehensive overview of the broader field is given, covering a variety of topics across multiple disciplines, including signal detection, cooperative sensing, machine learning, as well as security issues. Only a qualitative comparison of signal detection methods is given. Authors identify spectrum sensing hardware requirements as a future challenge and also note that published experiments to date mostly use energy detection. In our work we address this challenge by evaluating methods beyond energy detection. We also investigate requirements in respect to sampling rate and number of samples.

In [16], signal detection methods are classified based on the required knowledge of the source signal and noise power. The paper gives a thorough overview of the theoretical background, however it presents directly comparable simulation results only for some of the presented methods. The authors conclude that more tests of practical realizations of signal detectors are required. Our work can be seen as a contribution towards this goal. The experiments we performed contradict their finding that cyclostationary detectors require higher sampling rates and number of samples than other detectors.

In [10], blind signal detection methods are reduced to a common form. Probability of detection and computational complexity of the methods is compared in numerical simulation using a common set of parameters. Covariance-based detection is found to be very computationally complex, which is in contrast with our observations. Unlike in our work, cyclostationary detection is considered non-blind.

In [13], MATLAB simulations of energy detector with various thresholding methods, cyclostationary and matched filter detection are tested for probability of detection versus signal-to-noise ratio under AWGN and Rayleigh fading. Unlike in our work, covariance- and eigenvalue-based detection is not evaluated.

A review of spectrum sensing for applications in vehicular networks is available in [8]. Authors identify TVWS as a possible way of providing extra spectrum when dedicated channels get congested. They consider energy detection, matched filtering and cyclostationary detection in a qualitative comparison.

In general, qualitative comparisons based on unrelated evaluations of individual methods are problematic and can lead to erroneous conclusions. Results of such evaluations are rarely directly comparable due to plethora of different signals, parameters and environments used in the evaluations. For instance in [25], energy detection is experimentally evaluated using a wireless sensor network, including measurements of minimal detectable signal power. A sine wave and a 4 MHz QPSK signal were used. In [26], a 4 MHz QPSK signal with 64 MHz sampling rate is used to experimentally evaluate a cyclostationary detector. In [27], a different cyclostationary detector is evaluated with a simulated wireless microphone signal using 2048 samples and 12 MHz sampling rate. In [11], a simulated wireless microphone signal with 6 MHz sampling rate and an experimentally recorded digital TV waveform is used to compare covariance-based detectors and energy detection. In [28], a BPSK signal is used in a simulation to evaluate eigenvalue-based detectors.

Individual evaluations would also benefit from a methodology, such as the one proposed in this paper. In [10], authors discuss the often missing quantitative comparisons in publications of novel methods. Clearly defined steps can aid researchers in performing such comparisons and documenting their experiments to help with the reproducibility of published results. For instance, evaluation in [26] does not document the parameter *N*′ of the FAM method used for cyclostationary detection and the number of trials used to obtain probability estimates. In [29], a cyclostationary detector based on compressive sensing is experimentally evaluated, however signal models used in the evaluation are not presented and confidence estimates of the results are not given.

## 3 Methodology

**Fig 1:**
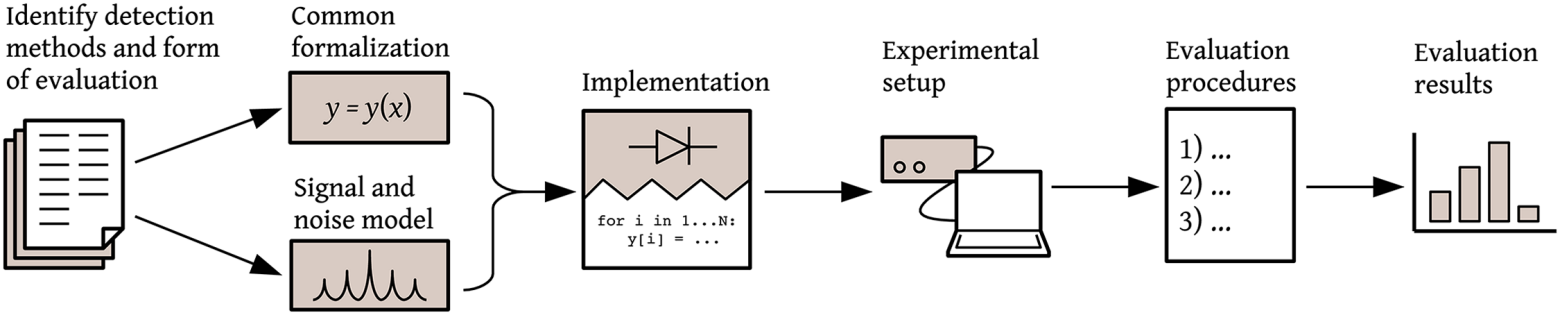
Overview of the proposed methodology for evaluating signal detection methods.

In this Section we propose a generic 7 step methodology that can be used for quantitatively evaluating and comparing all existing signal detection methods in simulation, lab test or both. By following the proposed methodology and documenting each step we also provide sufficient information to other efforts attempting to reproduce or extend our work. A graphical overview of the proposed methodology is presented in Fig 1.

### Step 1: Identify detection methods and form of evaluation

As a first step, one has to identify the target signal detection methods to be compared. For each method this includes finding the description, the formalization including any parameters, possibly a reference implementation and the performance evaluation carried out by the original author. The identified methods can then be evaluated against each other in a simulation environment, in an experimental lab test or both.

In this paper, we selected nine representative methods from four categories: energy detection, three covariance-based detection methods, four eigenvalue-based detection methods and one cyclostationary detector. We evaluated these methods in a lab test. We consider a scenario in which a TVWS device attempts to detect the presence of analog and digital legacy devices before transmitting.

### Step 2: Common formalization

In this step, the selected detection methods from Step 1 have to be brought to the same form. The goal is to document how a method establishes a relationship between the channel observation and the binary channel occupancy decision. The notations in the formulas have to be the same across all methods. At this point also parameters common to all methods are identified.

We represented all nine methods selected in the previous step as a scalar test statistic, which is a function of a vector of signal samples. We use a static threshold for the binary decision. We identified commonly used parameters such as time of sampling, target probability of false alarm, etc. This definition is detailed in Section 4. An example of alternative formalizations would be to have multiple features instead of a scalar test statistic or to use a different thresholding method.

### Step 3: Signal and noise model

In the third step, signal models that will be used to represent vacant and occupied channels have to be selected. The detection is performed using various levels of signal and noise power, which also have to be selected. This selection is affected by the assumptions about signal and noise in the methods. Some methods focus on detection of specific types of waveforms while others have more generic assumptions about signals being detected. The choice of models is also affected by the form of evaluation identified. In experimental evaluation, practical limitations (e.g. receiver noise) must be taken into account. Note that this step is relatively independent of the choice of method formalization in Step 2.

In this study we use a IEEE wireless microphone signal test vector, a IEEE 802.15.4 direct sequence spread spectrum BPSK waveform and Gaussian noise, as discussed in section 5. Common alternative signal models seen in the literature are BPSK [10, 13, 28] and QPSK [25, 26] modulated carriers using various settings and an unmodulated carrier [28].

### Step 4: Implementation

Next, the implementation details should be determined. This is useful for reproducibility, allowing other researchers to accurately and unambiguously repeat the results of the evaluation. In the case where software implementation is published as well, it also allows for code re-use. Furthermore, code additionally clarifies the signal detection methods used in case of any ambiguity in their mathematical description.

Implementation aspects for instance include the choice of receiver and signal generation hardware used in the lab test and description of any hardware signal processing components. Similarly for software components, detailed information on frameworks, libraries, languages and compilers is essential for rigorous evaluation. For the evaluation of computational complexity, methods should share as many software implementation details as possible for the results to be comparable.

In our evaluation, we implemented the nine selected methods in software, using a common *software-defined radio* (SDR) digital front-end as a receiver. Additionally, we implemented energy detection also with an analog energy detector. All software was written in an interpreted language due to its simplicity and speed of development. Signals were generated using a laboratory vector signal generator. The detailed description is provided in Section 6.

### Step 5: Experimental setup

In this step, the complete experimental setup should be documented. In case of a lab test, this includes the physical setup and any extra equipment used that was not already described in the previous step. Device settings that affect the outcome of the evaluation should be documented as well. Values for any parameters specific to detection methods used should be determined, as well as values for parameters common to all methods.

We use a table-top setup with components connected via a coaxial cable. We perform our evaluation in the UHF frequency band and select sampling rates and sensing times consistent with our chosen TVWS device scenario. We chose to evaluate covariance- and eigenvalue-based detectors with and without filter compensation and with 4 values for the smoothing factor. We evaluate cyclostationary detection with 2 values for *N*′. Detailed description is in section 7.

### Step 6: Evaluation procedures

The sixth step of our methodology requires documentation of evaluation procedures. This step should describe how the results of the evaluation were obtained using the setup described in the previous step.

We evaluate minimal detectable signal at a fixed probability of false alarm and minimal required probability of detection, noise level sensitivity and computational complexity. Detailed descriptions are in section 8.

### Step 7: Evaluation results

The final step of our methodology discusses the evaluation results. This step is relevant for checking the correctness and completeness of the results and to validate these against theoretical findings. The results should be presented with estimated confidence intervals that present uncertainties in measurements and statistical analysis. Our results are presented in section 9.

## 4 Common formalization of signal detection methods

A signal detector makes a binary decision based on the observed signal values on the channel during a limited time window. In analog implementations, the observed values can be represented as a continuous function *x*(*t*) defined for *t* ∈ [0, *t*_*s*_]. In digital implementations a more useful representation is a vector **x** of *N*_*s*_ observed signal samples in the time domain:

$$\begin{array}{c} x=\left[x_{n}\right]=\left\{x_{0},x_{1},\ldots x_{N_{s}-1}\right\} \end{array}$$ (1)

We consider only real valued signals in this paper.

The null hypothesis $H_{0}$ for the detector is that a channel is vacant. Alternative hypothesis $H_{1}$ is that the channel is occupied by another user.

In the case of a vacant channel it is assumed that the observed samples consist of only noise. In the case of an occupied channel, the observed samples contain noise in addition to some information-carrying signal *s*_*n*_. Various spectrum sensing methods make different assumptions about the properties of *s*_*n*_ to distinguish it from noise. Written formally [30]:

$$\begin{array}{c} \begin{array}{ccc} H_{0}:x_{n} & = & u_{n}\\ H_{1}:x_{n} & = & u_{n}+s_{n} \end{array}\;n\in \left[0,N_{s}-1\right] \end{array}$$ (2)

An event when the detector reports an occupied channel and $H_{1}$ is true is called a *correct detection*. Alternatively, a *false alarm* event happens if $H_{0}$ is true while the detector reports the occupied channel. Both of these events are assigned probabilities, *P*_*d*_ and *P*_*fa*_ respectively.

Probabilities are given in relation to the signal power:

$$\begin{array}{c} P_{in}=\frac{1}{N_{s}}\sum_{n=0}^{N_{s}-1}s_{n}^{2} \end{array}$$ (3)

A good spectrum sensing method will keep *P*_*d*_ high even for low *P*_*in*_ and short sensing time *N*_*s*_. In practical applications, to have a useful detection method, the probability of detection should stay above a certain threshold.

The signal detection methods considered in this paper operate by defining a scalar test statistic *γ* = *γ*(**x**) as a function of observed signal samples. A threshold *γ*_0_ is then defined, which is used to make the binary decision as follows:

$$\begin{array}{c} \begin{array}{ccc} H_{0} & \text{if} & \gamma \left(x\right)\leq \gamma_{0}\\ H_{1} & \text{if} & \gamma \left(x\right)>\gamma_{0} \end{array} \end{array}$$ (4)

**Fig 2:**
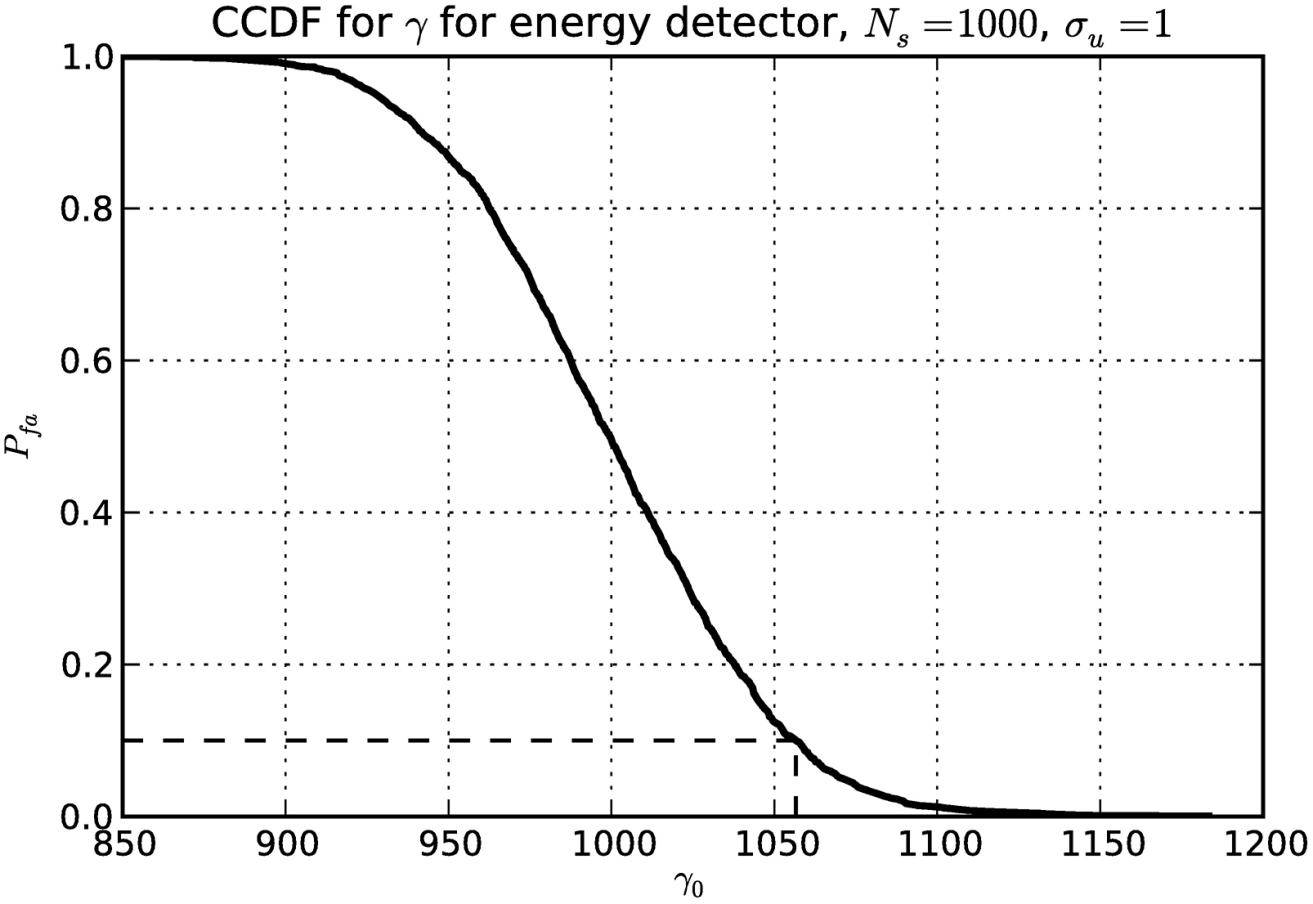
Complementary cumulative distribution function for energy detector test statistic *γ* obtained through numerical simulation of 2000 measurements. Dashed lines indicate the threshold value *γ*_0_ for a chosen *P*_*fa*_ = 0.1.

Threshold selection can be challenging. In our evaluation we used the empirical *constant false alarm rate* (CFAR) method [12]. A large number of signal sample vectors **x** was obtained for a known-empty channel. Using these measurements we approximated the complementary cumulative distribution function (CCDF) for *γ* in the case of $H_{0}$. The approximate *γ*_0_ for a desired probability of false alarm *P*_*fa*_ can then be read from the graph, as illustrated in Fig 2.

### 4.1 Energy detection

All radio transmitters by definition emit energy into the electromagnetic field. Energy detection (ED) is the simplest spectrum sensing method. As such it is often used as a baseline when evaluating other spectrum sensing methods. Energy detection is the optimal non-coherent detection algorithm [30].

The test statistic *γ* for energy detectors is based on the definition of signal energy, which is related to the physical concept of energy. For a continuous signal, using the common notation from this paper, the formalization is:

$$\begin{array}{c} \gamma =\int_{0}^{t_{s}}x^{2}\left(t\right)dt \end{array}$$ (5)

For sampled-time real signals, the equation transforms into:

$$\begin{array}{c} \gamma =\sum_{n=0}^{N_{s}-1}x_{n}^{2} \end{array}$$ (6)

The largest disadvantage of energy detection is the fact that the test statistic is as sensitive to noise power as to the signal power. In practice the noise power varies. Internal receiver noise changes with production tolerances, temperature and other environmental factors. Environment noise changes with time and location. This leads to a *SNR wall* [31], where a detector is incapable of reliably detecting a signal, regardless of the choice of *N*_*s*_, when signal power is small compared to noise power.

### 4.2 Covariance-based detection

Covariance methods attempt to solve the problem of unknown noise power by deriving the test statistic from sample covariance instead of variance (i.e. power). Covariance methods work on two basic assumptions: firstly, noise samples are independent and hence uncorrelated, and secondly, samples of realistic information-carrying signals are correlated to some degree [11].

Sample covariance estimates *σ*_*l*_ are calculated based on the vector of observed signal samples:

$$\begin{array}{c} \sigma_{l}=\frac{1}{N_{s}}\sum_{n=0}^{N_{s}-1}x_{n}\cdot x_{n-l}\;l\in \left[0,L-1\right] \end{array}$$ (7)

where *L* is a parameter called a *smoothing factor*, which determines the minimum time window within which the signal must exhibit correlation.

The test statistic is derived from a Toeplitz matrix of covariance estimates **R**.

$$\begin{array}{c} R=\left[r_{ij}\right]=\left[\begin{array}{cccc} \sigma_{0} & \sigma_{1} & \ldots & \sigma_{L-1}\\ \sigma_{1} & \sigma_{0} & \ldots & \sigma_{L-2}\\ \vdots & \vdots & \ddots & \vdots \\ \sigma_{L-1} & \sigma_{L-2} & \ldots & \sigma_{0} \end{array}\right] \end{array}$$ (8)

In general, non-diagonal elements of **R** will be near zero in case of a vacant channel while diagonal elements are related to signal power and are not affected by correlations in the signal. Covariance methods calculate a scalar test statistic based on this observation:

$$\begin{array}{c} \gamma =\frac{T_{1}}{T_{2}} \end{array}$$ (9)

where *T*_1_ is related to non-diagonal elements of **R** and *T*_2_ to diagonal elements. We tested several variations on this method with different definitions of these two variables. They are listed in Table 1.

**Table 1:** Covariance-based test statistics.

Covariance Absolute Value (CAV) [11]	$T_{1}=\frac{1}{L}\sum_{i=1}^{L}\sum_{j=1}^{L}\left|r_{ij}\right|$	$T_{2}=\frac{1}{L}\sum_{i=1}^{L}\left|r_{ii}\right|$
Maximum Auto-correlation (MAC) [28]	$T_{1}=\frac{1}{L}\max_{i=1}^{L}\max_{j=1}^{L}\left|r_{ij}\right|$	$T_{2}=\frac{1}{L}\sum_{i=1}^{L}\left|r_{ii}\right|$
Covariance Frobenius Norm (CFN) [32]	$T_{1}=\frac{1}{L}\sum_{i=1}^{L}\sum_{j=1}^{L}{\left|r_{ij}\right|}^{2}$	$T_{2}=\frac{1}{L}\sum_{i=1}^{L}{\left|r_{ii}\right|}^{2}$

While covariance-based methods are robust against changes in power of Gaussian noise, realistic signals also include noise samples that are not independent. This so-called *noise coloring* can be due to narrow-band filtering in the receiver analog front-end [11] or non-white natural and man-made external noise [33]. The sensitivity of covariance-based methods in practical scenarios is hence limited by the noise-coloring uncertainty.

Noise coloring due to time-invariant filtering can be compensated by using a transformed covariance matrix **R**′ when calculating test statistics [11]:

$$\begin{array}{c} R^{\prime }=Q^{-1}RQ^{-1} \end{array}$$ (10)

where **Q** can be pre-calculated for a known receiver from the covariance-matrix of noise-only samples **R**_**n**_ using the matrix square root:

$$\begin{array}{c} Q=R_{n}^{\frac{1}{2}} \end{array}$$ (11)

### 4.3 Eigenvalue-based detection

Eigenvalue-based detection methods are closely related to covariance-based detection. These methods attempt to trade off some of the simplicity for better signal detection performance [34]. Here, the test statistic is based on eigenvalues λ_*l*_ of the covariance matrix **R**, ordered by their absolute value:

$$\begin{array}{c} |\lambda_{0}\left|\geq \right|\lambda_{1}\left|\geq \ldots \geq \right|\lambda_{L-1}| \end{array}$$ (12)

Eigenvalue calculation requires on the order of *L*^3^ operations, however since *L* is typically small compared to *N*_*s*_, the calculation of covariance estimates in (7) dominates computing time [34]. Several variations of Eigenvalue-based test statistics are listed in Table 2.

**Table 2:** Eigenvalue-based test statistics.

Maximum-minimum Eigenvalue (MME) [34]	$\gamma =\frac{|\lambda_{0}|}{|\lambda_{L-1}|}$
Arithmetic to Geometric Mean (AGM) [28]	$\gamma =\frac{\frac{1}{L}\sum_{l=0}^{L-1}\left|\lambda_{l}\right|}{\sqrt[{L}]{\prod_{l=0}^{L-1}\left|\lambda_{l}\right|}}$
Maximum Eigenvalue to Trace (MET) [28]	$\gamma =\frac{|\lambda_{0}|}{\sum_{l=0}^{L-1}\lambda_{l}}$
Energy to Minimum Eigenvalue (EME) [34]	$\gamma =\frac{\sum_{n=0}^{N_{s}-1}x_{n}^{2}}{\lambda_{L-1}}$

Noise coloring compensation method in (10) can be applied to eigenvalue-based detection as well.

### 4.4 Cyclostationary detection

Cyclostationary detectors exploit the fact that practical information carrying signals often have a repeating structure over some length of time [35]. Such cycles can arise from the constant bit or symbol rate in digital transmissions or from constant carrier tones in analog transmissions. This is in contrast to noise, which is assumed to be a stationary signal.

In this paper, we based the cyclostationary detection on the commonly used *spectral correlation function* *S*_*x*_(*α*, *f*):

$$\begin{array}{c} R_{x}\left(\alpha ,\tau \right)=\lim_{T\to \infty }\frac{1}{T}\int_{-\frac{T}{2}}^{\frac{T}{2}}x\left(t+\frac{\tau }{2}\right)x\left(t-\frac{\tau }{2}\right)e^{-i2\pi \alpha t}dt \end{array}$$ (13)

$$\begin{array}{c} S_{x}\left(\alpha ,f\right)=\int_{-\infty }^{\infty }R_{x}\left(\alpha ,\tau \right)e^{-i2\pi f\tau }d\tau \end{array}$$ (14)

where *α* is the *cyclic frequency* and *R*_*x*_ is the *cyclic correlation function*.

It can be seen that *S*_*x*_(*α*, *f*) will be zero for *α* ≠ 0 for stationary noise. On the other hand, a cyclostationary signal will have *S*_*x*_(*α*, *f*) non-zero for specific values of *α* and *f*.

We approximate *S*_*x*_(*α*, *f*) for its complete range of *α* ∈ [−*f*_*s*_, *f*_*s*_] and $f\in [-\frac{f_{s}}{2},\frac{f_{s}}{2}]$. This makes our detector sensitive to a wide range of cyclostationary signals. This approach overcomes a known shortcoming with detectors that assume a known *α* where sampling rate uncertainties affect the detector sensitivity [26].

**Fig 3:**
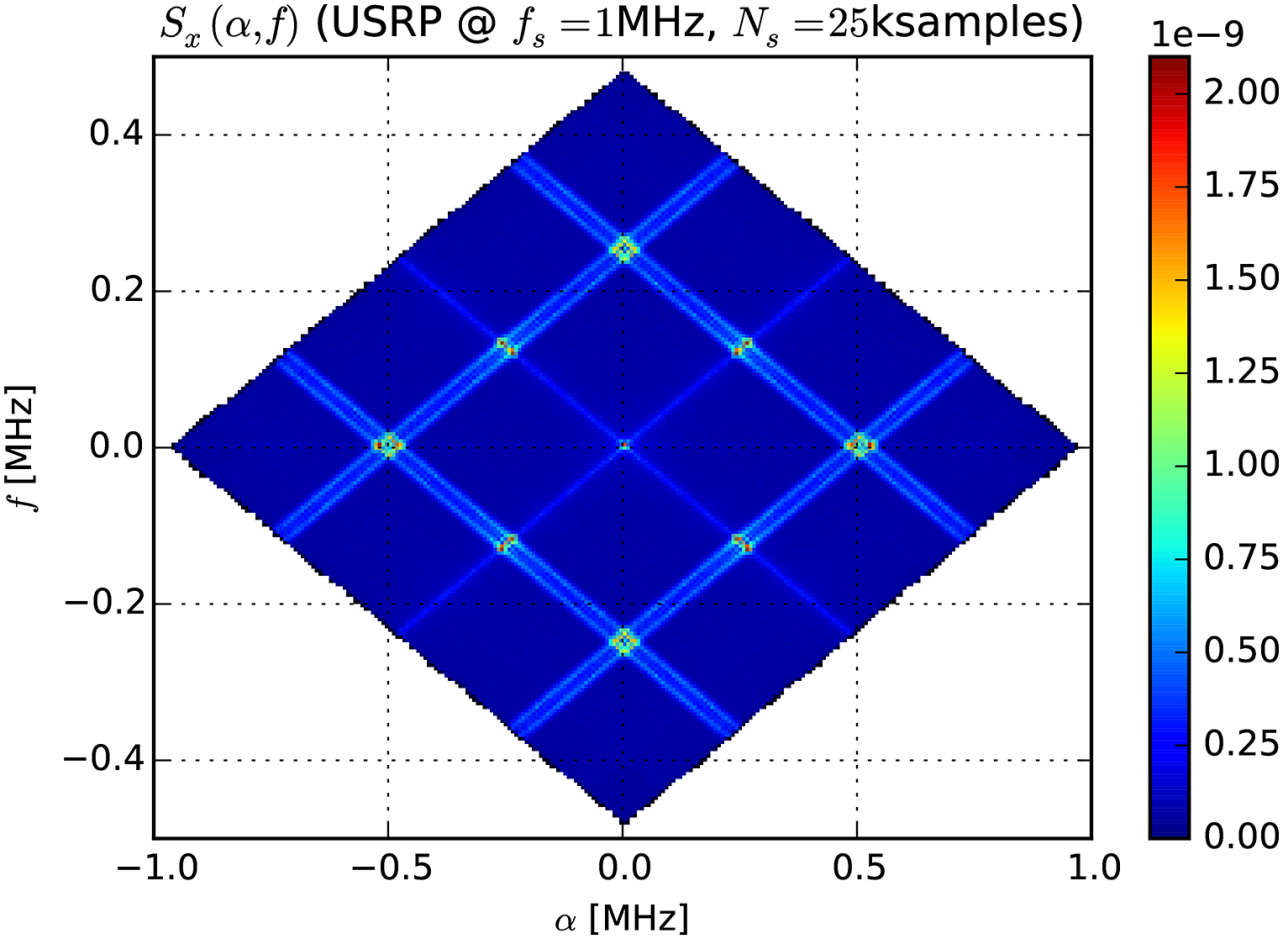
Spectral correlation function for a frequency modulated carrier (19), approximated using the FAM method from USRP measurements (*N*′ = 256).

We use the *FFT accumulation method* (FAM) [36] to approximate the spectral correlation function. FAM is based on sets of conventional Fourier transforms. An example plot of *S*_*x*_(*α*, *f*) approximation showing characteristic diamond shapes is shown in Fig 3.

The FAM method takes several parameters. *N*′ is the size of the first FFT and determines the frequency resolution Δ*f*, *L* is the decimation factor (also called smoothing factor and usually set to $\frac{N^{\prime }}{4}$) and $P\approx \frac{N_{s}}{L}$ is the size of the FFTs in the second set and determines the cyclic frequency resolution Δ*α*.

$$\begin{array}{ccc} \Delta f & = & \frac{f_{s}}{N^{\prime }} \end{array}$$ (15)

$$\begin{array}{ccc} \Delta \alpha & = & \frac{f_{s}}{PL}\approx \frac{f_{s}}{N_{s}} \end{array}$$ (16)

In our experiment we use the following test statistic that has been previously applied to wireless microphone detection [27]:

$$\begin{array}{c} \gamma =\max_{f,\alpha }\frac{S_{x}\left(\alpha ,f\right)}{S_{x}\left(0,f\right)}\;\alpha \neq 0 \end{array}$$ (17)

In respect to computational complexity, the dominating part of the FAM method is the calculation of the second set of Fourier transforms. This step requires *N*′^2^ complex *P*-point transforms which in turn require 2*N*′^2^ *P* log_2_ *P* floating point multiplications [36].

## 5 Signal and noise model selection

As models of the signals to be detected by the methods under evaluation, we selected one of the IEEE wireless microphone signal test vectors [37] and the direct sequence spread spectrum BPSK waveform used as one of the physical layers in the IEEE 802.15.4 standard [38]. We considered these two test vectors as representative of common analog and digital transmissions present in the sub-1 GHz part of the radio spectrum.

### 5.1 Microphone signal

The IEEE wireless microphone test vectors are designed to emulate transmissions from legacy analog UHF wireless microphones, also called *programme making and special events* (PMSE) devices. In contrast to transmissions from real microphones that vary with audio input levels, these vectors were defined to provide deterministic waveforms that can be reliably reproduced in simulations and in laboratory spectrum sensing experiments. They can be reproduced using commonly available equipment. These test vectors are often used in the literature for signal detection evaluations [12, 27].

In [38], 6 different wireless microphone test vectors are defined. The document recommends that spectrum sensing schemes in the context of the IEEE 802.22 standard are evaluated using all 6 vectors. However due to limited time and a lack of a Rayleigh fading simulator we chose to perform our evaluation using only the “Outdoor, LOS, soft speaker” vector (same vector was used in [27]). Since our task was to perform a scientific evaluation and not evaluate a specific engineering solution for IEEE 802.22 networks, we considered one vector sufficient. Implementations for the other microphone test vectors are available in our source code repository (see Section 10).

The selected microphone test vector is based on a constant-amplitude, frequency modulated carrier using a single tone as a modulating signal. In an analog form it can be written as:

$$\begin{array}{c} s_{mic}\left(t\right)=A\cos\left(2\pi f_{c}t+\frac{f_{dev}}{f_{m}}\cos\left(2\pi f_{m}t\right)\right) \end{array}$$ (18)

where *A* is the signal amplitude, *f*_*c*_ is the carrier frequency, *f*_*m*_ is the frequency of the modulating tone and *f*_*dev*_ is the frequency deviation. The soft speaker vector defines the following values (see Section 3, paragraph 2 in [38]):

$$\begin{array}{c} f_{m}=3.9\text{kHz}\;f_{dev}=15.0\text{kHz} \end{array}$$ (19)

### 5.2 BPSK signal

The direct sequence spread spectrum BPSK waveform is one of the physical layers used by IEEE 802.15.4 low-rate wireless personal area networks in the sub 1-GHz spectrum. Our model follows the specification given in Section 11.2 of [38]. A sequence of binary symbols to be transmitted is first converted to a binary chip sequence using a static symbol-to-chip mapping table (see Table 78 in [38]). Each symbol is converted into a sequence of 15 chips.

Individual chips are transmitted as a raised cosine pulse with the roll-off factor of 1. The single pulse signal *p*(*t*) can be written as:

$$\begin{array}{c} p\left(t\right)=\{\begin{array}{ccc} 1 & \text{if} & t^{\prime }=0,\\ 0.5 & \text{if} & t^{\prime }=\pm 0.5,\\ \frac{\sin\pi t^{\prime }}{\pi t^{\prime }}\frac{\cos\pi t^{\prime }}{1-4{t^{\prime }}^{2}} & \text{otherwise.} \end{array} \end{array}$$ (20)

$$\begin{array}{c} t^{\prime }=\frac{t}{T_{c}} \end{array}$$ (21)

where *T*_*c*_ is the chip period. The complete test vector can be written as a sum of pulses with pulse polarity depending on the chip value:

$$\begin{array}{c} s_{bpsk}\left(t\right)=A\cos\left(2\pi f_{c}t\right)\sum_{n=0}^{N_{c}}\left(2c_{n}-1\right)p\left(t-nT_{c}\right) \end{array}$$ (22)

where *A* is the signal amplitude, *f*_*c*_ is the carrier frequency, *N*_*c*_ is number of chips in the test vector and *c*_*n*_ ∈ [0, 1] is the binary value of the n-th chip in the vector. For our evaluation we chose the chip rate specified for operation in the 868 MHz band:

$$\begin{array}{c} T_{c}=\frac{1}{300000}\text{s}\approx 3.3\mu \text{s} \end{array}$$ (23)

### 5.3 Noise

In our evaluation, two noise signals were added to the selected test signal:

$$\begin{array}{c} x\left(t\right)=s\left(t\right)+u_{int}\left(t\right)+u_{ext}\left(t\right) \end{array}$$ (24)

*u*_*int*_ is the unavoidable noise generated by the equipment used in the experiment: internal noise of the receiver and the signal generator. The characteristics of this noise are defined by the construction of devices used in the experiment. We considered it colored and non-Gaussian.

*u*_*ext*_ is the external noise generated by the signal generator. This noise was zero-mean Gaussian with i.i.d. samples:

$$\begin{array}{c} u_{ext}\left(t\right)∼N\left(0,\sigma_{u}^{2}\right) \end{array}$$ (25)

where $\sigma_{u}^{2}$ is the selected noise power.

We selected Gaussian noise because all detection methods in our evaluation, except for energy detection, aim to be immune to changes in Gaussian noise power. By selecting this type of noise, we wanted to focus on this specific property of the methods, rather than on their robustness to more realistic sources of external noise. The latter could be a focus of a separate study. Furthermore, it should be noted that while the external noise model *u*_*ext*_ was Gaussian, the actual noise produced in our experiment was not Gaussian due to the practical limitations of our equipment, such as the limited signal generator bandwidth and waveform length.

## 6 Implementation

### 6.1 Receiver hardware

In our evaluation we used two radio receivers: An Ettus Research USRP N200 device and an in-house developed SNE-ISMTV-UHF receiver. These devices provided raw data that was further processed in software.

*Ettus Research USRP N200 device* is a software-defined radio front-end [39]. The USRP series of devices commonly appears in the literature due to relatively low cost and high performance. It was chosen as a typical representative of a receiver used in a software-defined radio architecture.

USRP device in our evaluation was equipped with an SBX daughterboard and could record the baseband signal using 14-bit complex (I/Q) samples. It has an adjustable receive bandwidth and a sampling frequency *f*_*s*_*USRP*__ up to 50 Msample/s [39]. We used the GNU Radio framework to control the device from a personal computer [40].

In our experimental evaluation we used the USRP only as a digital receiver to record vectors of *N*_*s*_ unprocessed baseband signal samples. These vectors were then passed to our software implementation of (digital) energy detection, covariance, eigenvalue and cyclostationary methods as described in Section 6.3.

*SNE-ISMTV-UHF receiver* is a VHF/UHF band receiver with an analog energy detector [41] based on the VESNA sensor node platform [42]. The receiver has a configurable channel filter bandwidth and contains an analog logarithmic power detector [43]. The output of the power detector is sampled by a 12-bit ADC with a fixed sampling rate *f*_*s*_*ISMTV*__ = 147kHz. VESNA spectrum sensor application was used to record the data from the sensor [44].

Of the detection methods listed in Section 4, only energy detection can be practically implemented in the analog domain. Analog energy detectors are common in practical spectrum sensing experiments due to their simplicity, low power usage and low cost of implementation. In our experimental evaluation we used the SNE-ISMTV-UHF receiver to implement analog energy detection.

Since the analog detector sampling rate was fixed with this hardware, we implemented different sensing times *t*_*s*_ required by our evaluation by summing a number of consecutive detector samples in software. Summation was done in linear scale. Hence, to arrive to the test statistic *γ* approximating (5), we used the following expression:

$$\begin{array}{c} \gamma =\sum_{n=0}^{N_{s}-1}K_{1}10^{K_{2}y_{n}} \end{array}$$ (26)

where *N*_*s*_ is the number of detector samples, *K*_1_ and *K*_2_ are detector scaling constants and *y*_*n*_ is the *n*-th detector sample. *N*_*s*_ was calculated from the detector sampling rate:

$$\begin{array}{c} N_{s}=\frac{t_{s}}{f_{s_{\text{ISMTV}}}} \end{array}$$ (27)

Scaling constants were chosen such that the value of *γ* was within the floating point range used in the calculation.

### 6.2 Signal and noise generation

All signals in our experiment were generated using the Rohde&Schwarz SMBV100A vector signal generator [45].

The IEEE wireless microphone signal test vector was implemented using the analog modulation block. The block was set for frequency modulation. Modulating signal was internal sine wave source set to parameters given in (19).

The IEEE 802.15.4 direct sequence spread spectrum BPSK waveform and the Gaussian noise signal were implemented using the arbitrary waveform (ARB) block of the signal generator. The block was set to sampling frequency *f*_*s*_*DAC*__. A vector of *N*_*s*_*DAC*__ I/Q samples was uploaded to the signal generator before the start of the experiment. The signal generator operated by continuously repeating the uploaded vector.

The I/Q vectors were generated in software on a personal computer. For the BPSK waveform, the symbol sequence entering the modulator was generated pseudo-randomly with a uniform distribution. Since the symbol sequence was random, the differential encoder specified in IEEE 802.15.4 was not implemented. Signal amplitude was full DAC range [−1, +1]. For the Gaussian noise signal, I/Q samples were similarly generated using a pseudo-random generator with a normal sample distribution. Standard deviation of digital samples was fixed at 0.19 to prevent excessive clipping. In both cases, the power of the actual signals was set by adjusting signal generator’s analog output level.

For noise signal, *f*_*s*_*DAC*__ was selected to be sufficiently above the sampling rate of receivers so that noise samples appeared uncorrelated despite the finite bandwidth of the signal source. Similarly, *N*_*s*_*DAC*__ was chosen to be large enough compared to the smoothing factor used in covariance- and eigenvalue-based detection:

$$\begin{array}{c} \frac{N_{s_{DAC}}}{f_{s_{DAC}}}=t_{p_{DAC}}\gg \Delta t=\frac{L}{f_{s_{USRP}}} \end{array}$$ (28)

where *t*_*p*_*DAC*__ is the period of the signal generated by ARB, Δ*t* is the time lag corresponding to the smoothing factor *L*.

### 6.3 Implementation of the methods

All software used in this evaluation was developed in the Python programming language (version 2.7). This choice was motivated by our knowledge of the language, a large set of available libraries for numeric calculation and good integration with both GNU Radio and VESNA libraries.

The software developed for this evaluation includes measurement automation, calculation of test statistics and data post-processing.

Measurements were fully automated. The experiment was controlled by a Python script running on a laptop PC. It recorded raw data from radio receivers to laptop’s hard drive and also controlled the signal generator according to the steps discussed in Section 8. The only manual intervention required was re-connecting the coaxial cable connection when changing the receiver.

The test statistics for energy detection, covariance, eigenvalue and cyclostationary detection discussed in Section 4 were also calculated from raw data using Python. We used NumPy and SciPy packages for fast vector operations and common mathematical functions [46].

When evaluating computational complexity of test statistic calculation, the Python interpreter was running on an Intel Xeon E5520 processor with 2.27 GHz CPU clock under GNU/Linux operating system. These benchmarks were using CPython 2.7.3 interpreter with NumPy 1.6.1 module.

Postprocessing, such as deriving metrics presented in Section 9, was performed using iPython notebooks.

The source code of Python scripts, as well as raw measurement data and iPython notebooks with calculations used in the evaluation are available on-line (https://github.com/avian2/spectrum-sensing-methods) enabling reproducibility and code re-use.

## 7 Experimental setup

**Fig 4:**
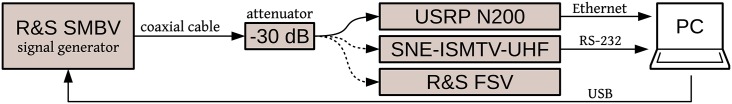
A diagram of our experimental setup. Coaxial cable with the attached attenuator was manually moved between devices. R&S FSV signal analyzer was used in power measurement mode to measure exact signal attenuation.

**Fig 5:**
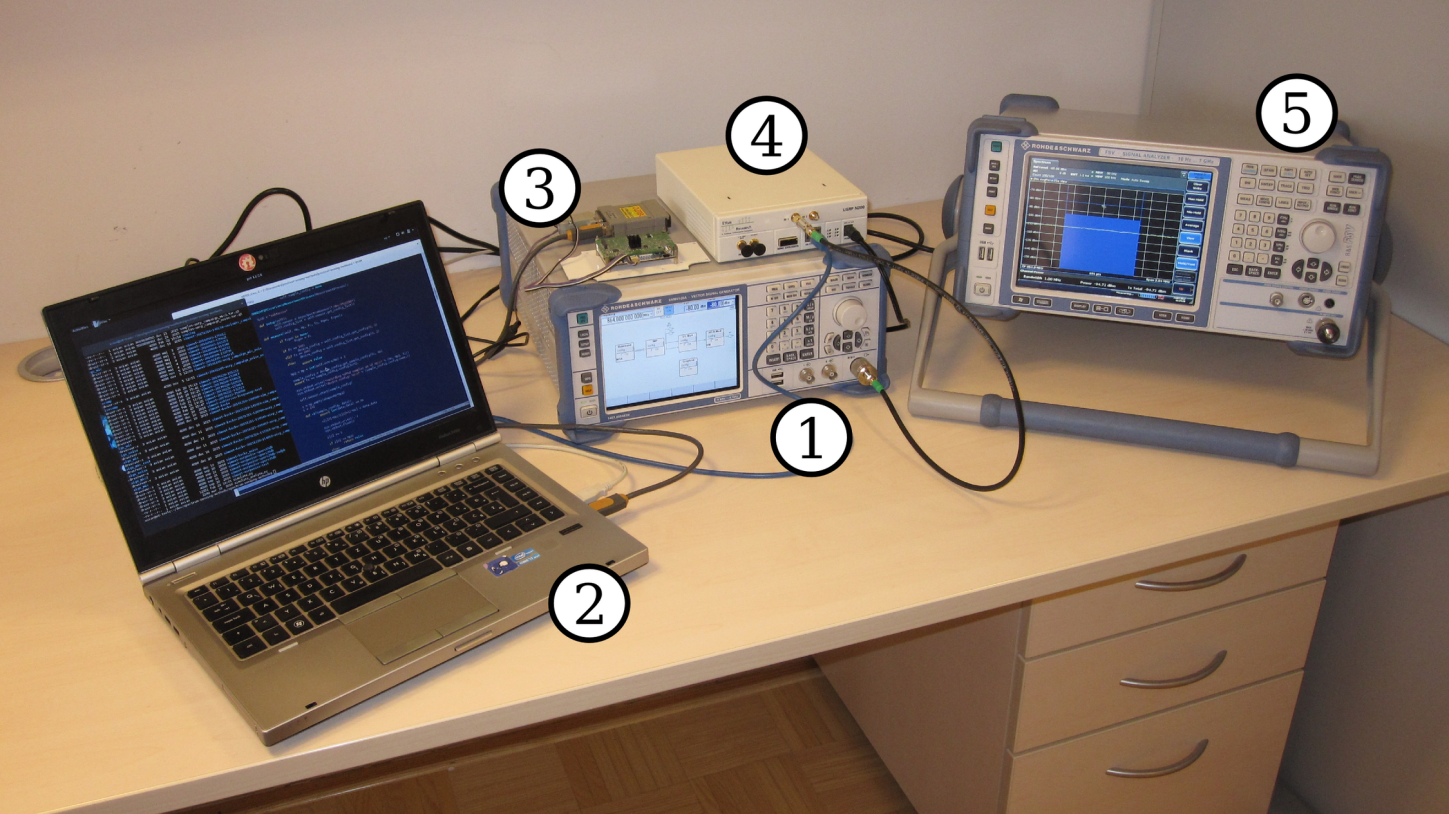
A photograph of our setup. (1) Signal generator, (2) laptop PC, (3) SNE-ISMTV-UHF receiver, (4) USRP N200 device, (5) signal analyzer.

Our laboratory setup is schematically presented in Fig 4. A photograph is shown in Fig 5. The output of the vector signal generator was connected to one of the radio receivers. A short length of low-loss 50*Ω* coaxial cable was used. We used a cable instead of an antenna to minimize interference from external sources. To protect the receivers from accidental overload, a 30 dB attenuator (Minicircuits VAT-30+) was added to the receiver end of the cable.

The USRP receiver was connected to the laptop PC controlling the experiment using Gigabit Ethernet. The SNE-ISMTV-UHF receiver was connected using a RS-232 serial interface. The signal generator was controlled by the measurement script on the laptop over a USB cable. Before starting the evaluation, the coaxial cable was connected to a Rohde&Schwarz FSV signal analyzer to measure total signal attenuation between the signal generator and receivers.

Before starting evaluations, all instruments were turned on for 2 hours to reach thermal equilibrium. The coaxial cable was connected to a calibrated Rohde&Schwarz FSV signal analyzer, configured as a power meter, to measure total signal attenuation in the cable and connectors. This measurement was required to accurately determine input signal power during the later steps in the experimental evaluation. The total measured signal attenuation between signal generator output and receiver input was *A* = 32.1dB for the minimal detectable signal measurements and *A* = 31.5dB for noise level sensitivity measurements.

After this initial step, the cable was connected to the USRP device, to evaluate the digital implementation of the detection methods discussed in Section 6. For the covariance-based and eigenvalue-based methods (Tables 1 and 2) we used four values for the smoothing factor *L* ∈ {5, 10, 15, 20}. Filter compensation was used for the evaluation described in Section 9.3. To evaluate cyclostationary detection we used the test statistic from (17) based on the spectral correlation function (SCF). We used two values of *N*′ ∈ {64, 128}.

After all digital signal detection implementations were evaluated, the cable was connected to the SNE-ISMTV-UHF device, to evaluate the analog implementation of energy detection in a similar way.

Code benchmarks discussed in Section 8.4 were performed on a different computer, with no other equipment connected. The benchmarks were using the same Python implementation of test statistics as those used for other evaluations.

### 7.1 Device settings

The central frequency for the signal generator and the USRP receiver was set to *f*_*c*_ = 864MHz. This frequency corresponds to the channel reserved for unlicensed use of wireless microphones in Europe. For measurements done with SNE-ISMTV-UHF, *f*_*c*_ = 850MHz was used, which was the closest frequency supported by this device.

We chose three settings for the devices, shown in Table 3, representing different classes of sensing devices. Settings A and B represent capabilities of an embedded sensing device. The required sample buffer length and sample rate can be implemented with microcontrollers with integrated A/D converters currently on the market [47]. Even though a simpler radio front-end than the USRP would most likely be employed in such a device, measurements using these two settings give an estimate of the performance of sensing methods achievable on low-cost embedded systems. Lastly, setting C was chosen to test performance that could be expected from a high-end device. Therefore a higher sampling rate and a longer receive buffer have been used.

**Table 3:** Device settings.

	*t*_*s*_[ms]	USRP		SNE-ISMTV-UHF	
		*f*_*s*_[kHz]	*N*_*s*_[samples]	*f*_*s*_[kHz]	*N*_*s*_[samples]
A	25.0	1,000	25,000	147	3,676
B	12.5	2,000	25,000	147	1,838
C	10.0	10,000	100,000	147	1,471

SNE-ISMTV-UHF device was configured to use a 1.7 MHz analog channel filter. To obtain energy detection results from SNE-ISMTV-UHF that would be comparable to USRP measurements, we adjusted the number of detector samples so that the total sensing time was equal to that of USRP measurements.

$$\begin{array}{c} N_{s_{\text{ISMTV}}}=t_{s}f_{s_{\text{ISMTV}}}=\frac{N_{s_{\text{USRP}}}}{f_{s_{\text{USRP}}}}f_{s_{\text{ISMTV}}} \end{array}$$ (29)

The Rohde&Schwarz SMBV100A vector signal generator was configured to generate the signal according to the model from Section 5. The following ARB settings were used for BPSK waveform generation:

$$\begin{array}{c} f_{s_{\text{DAC}-\text{BPSK}}}=18\text{MHz}\;N_{s_{\text{DAC}-\text{BPSK}}}=600\text{ksamples} \end{array}$$ (30)

The following ARB settings were used for noise generation:

$$\begin{array}{c} f_{s_{\text{DAC}-\text{noise}}}=50\text{MHz}\;N_{s_{\text{DAC}-\text{noise}}}=500\text{ksamples} \end{array}$$ (31)

## 8 Evaluation procedures

### 8.1 Comparing minimal detectable signal

In our first experiment, we compared the minimal signal power that was detectable using a given hardware receiver, sensing method and test signal.

With the signal generator turned off we first obtained *N*_*p*_ = 1000 samples of the test statistic *γ* for each method. Then, using the method illustrated in Fig 2, we estimated the threshold *γ*_0_ for the chosen probability of false alarm *P*_*fa*_ = 0.1.

With the signal generator turned on, set to output one of our test signals and output power *P*_*g*_, we then obtained another set of *N*_*p*_ samples of the test statistic *γ* for each method. By comparing these samples to the chosen threshold, we estimated the probability of detection *P*_*d*_ for input power *P*_*in*_. This procedure was repeated for each test signal.

We used the maximum likelihood estimation to estimate *P*_*d*_ from the *N*_*p*_ detection trials. To verify that the number of trials was sufficient for an accurate estimate, we also calculated the binomial confidence interval for *P*_*d*_ using the Clopper-Pearson method [48]. We chose this method because it gives accurate results when the estimated probability is close to zero or one (which was the case in our experiments) and because its implementation was readily available to us. In this verification we only considered the binomial detection process and assumed that the uncertainty of *γ*_0_ determination did not significantly affect the confidence interval of the final result. We also did not account for uncertainties in devices, such as errors in calibration, temperature changes, uncertainties in connector attenuations, and so on. We assumed these effects on our results were minimal.

Input power *P*_*in*_ was calculated as:

$$\begin{array}{c} P_{in}=P_{g}-A \end{array}$$ (32)

where *A* is the measured attenuation between the signal generator output and receiver input. We recorded *P*_*d*_ for *P*_*g*_ between -100 dBm and -70 dBm. We considered a signal reliably detected if *P*_*d*_ > 0.9.

### 8.2 Comparing noise level sensitivity

To compare the sensitivity of the methods to differences in noise level, we used the signal generator to inject additional noise into the antenna connector of USRP receiver (SNE-ISMTV-UHF was not used in this part of the experiment).

With the signal generator turned on, set to Gaussian noise and output power *P*_*g*_, we obtained a set of *N*_*p*_ samples of the test statistic *γ* for each method. By comparing *γ* samples to the threshold from the previous experiment, we estimated the probability of false alarm *P*_*fa*_ for change in noise power Δ*P*_*noise*_.

$$\begin{array}{c} \Delta P_{\text{noise}}=\frac{P_{n}}{P_{g}-A} \end{array}$$ (33)

where *P*_*n*_ is receiver’s internal noise power which was estimated using energy detection with the signal generator turned off.

We recorded *P*_*fa*_ for *P*_*g*_ between -110 dBm and -80 dBm. We considered that the noise floor change affected the detection when *P*_*fa*_ ≥ 0.2. This is twice the initially set value for *P*_*fa*_ used for the estimation of the threshold *γ*_0_.

### 8.3 Effect of noise compensation

To evaluate the effect of noise compensation on the performance of covariance-based methods, we repeated the minimal detectable signal and noise level sensitivity experiments with the compensation method applied. We applied the time-invariant filter compensation in (10) on the covariance matrix before the calculation of the test statistics *γ*. Noise-only covariance matrix **R**_**n**_ was obtained from *N*_*s*_ samples, obtained when the signal generator was turned off.

### 8.4 Comparing computational complexity

To measure the *γ* function execution time, we used the *timeit* module from the Python standard library. As an input we used *N*_*s*_ = 25ksamples of Gaussian noise samples. For each function we did 10 campaigns of *N*_*p*_ = 1000 repetitions. We took the time from the fastest campaign as the relevant result and discarded the rest. This minimized the effect of other processes interfering with the measurement [49].

## 9 Evaluation results

### 9.1 Comparing minimal detectable signal

**Fig 6:**
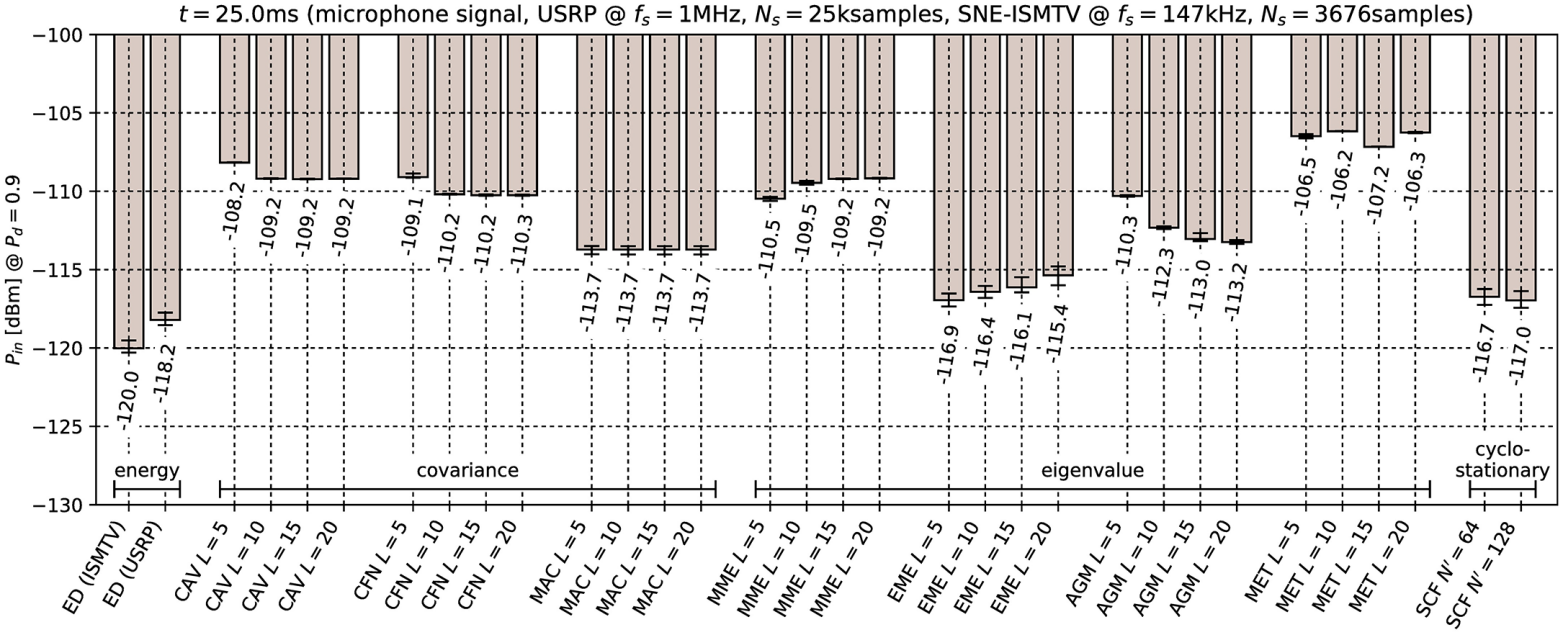
Lowest detectable power *P*_*in*_ of the microphone signal for various methods with sensing time *t* = 25.0ms. Error bars show binomial confidence intervals for *α* = 10^−6^.

**Fig 7:**
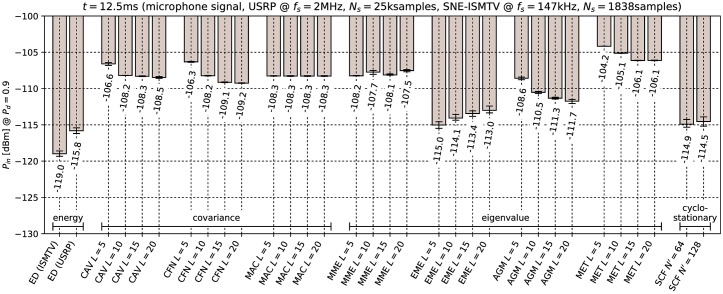
Lowest detectable power *P*_*in*_ of the microphone signal for various methods with sensing time *t* = 12.5ms. Error bars show binomial confidence intervals for *α* = 10^−6^.

**Fig 8:**
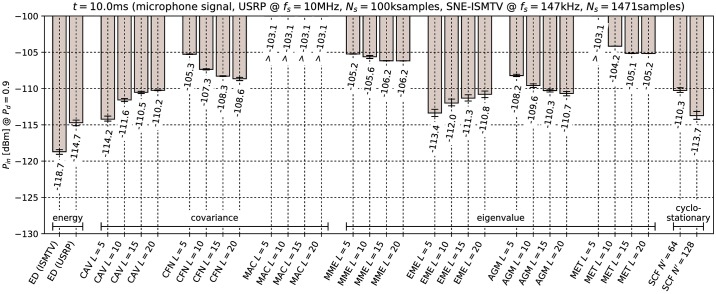
Lowest detectable power *P*_*in*_ of the microphone signal for various methods with sensing time *t* = 10.0ms. Error bars show binomial confidence intervals for *α* = 10^−6^.

**Fig 9:**
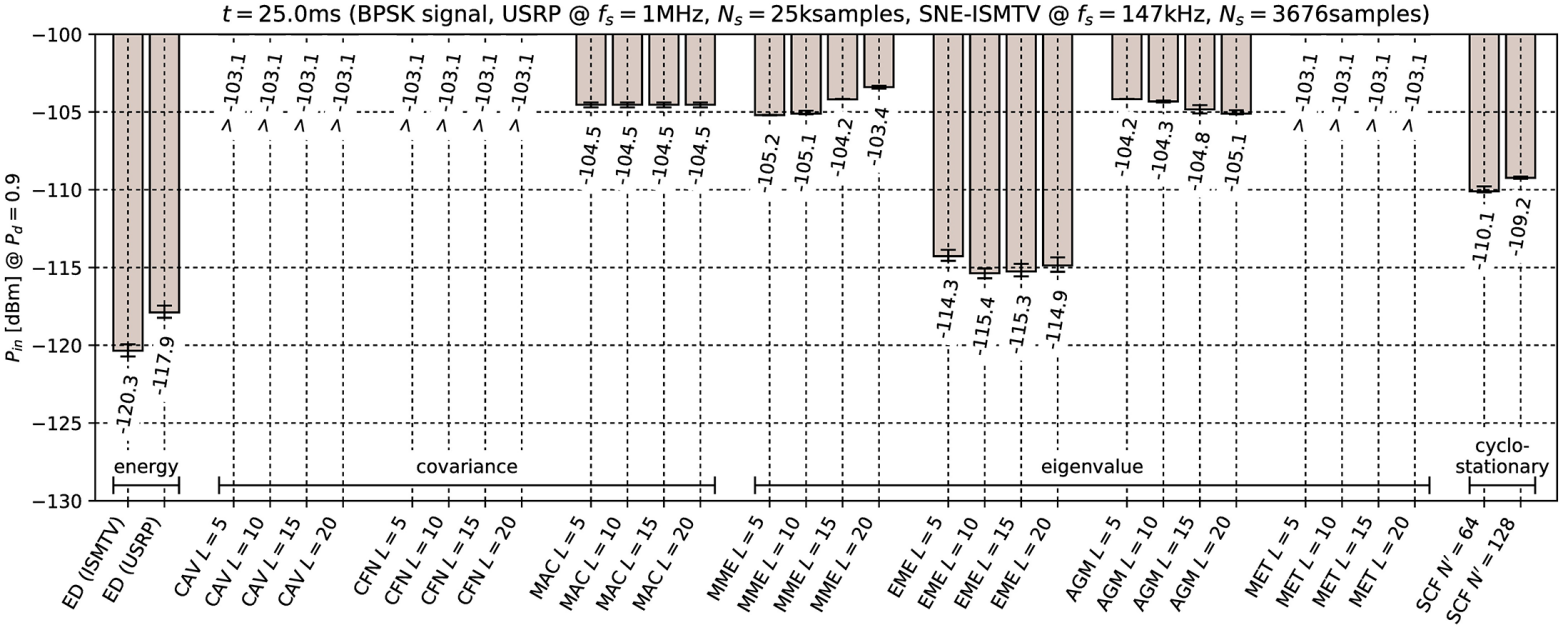
Lowest detectable power *P*_*in*_ of the BPSK signal for various methods with sensing time *t* = 25.0ms. Error bars show binomial confidence intervals for *α* = 10^−6^.

**Fig 10:**
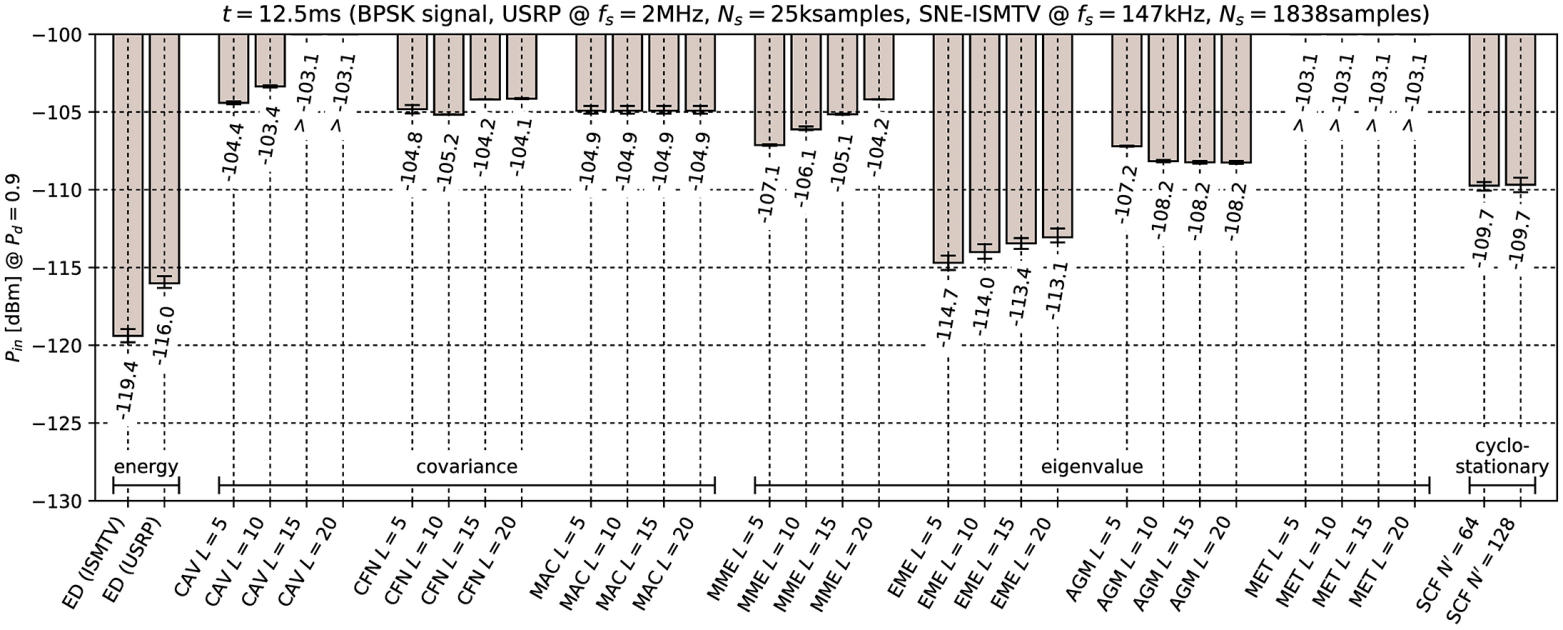
Lowest detectable power *P*_*in*_ of the BPSK signal for various methods with sensing time *t* = 12.5ms. Error bars show binomial confidence intervals for *α* = 10^−6^.

**Fig 11:**
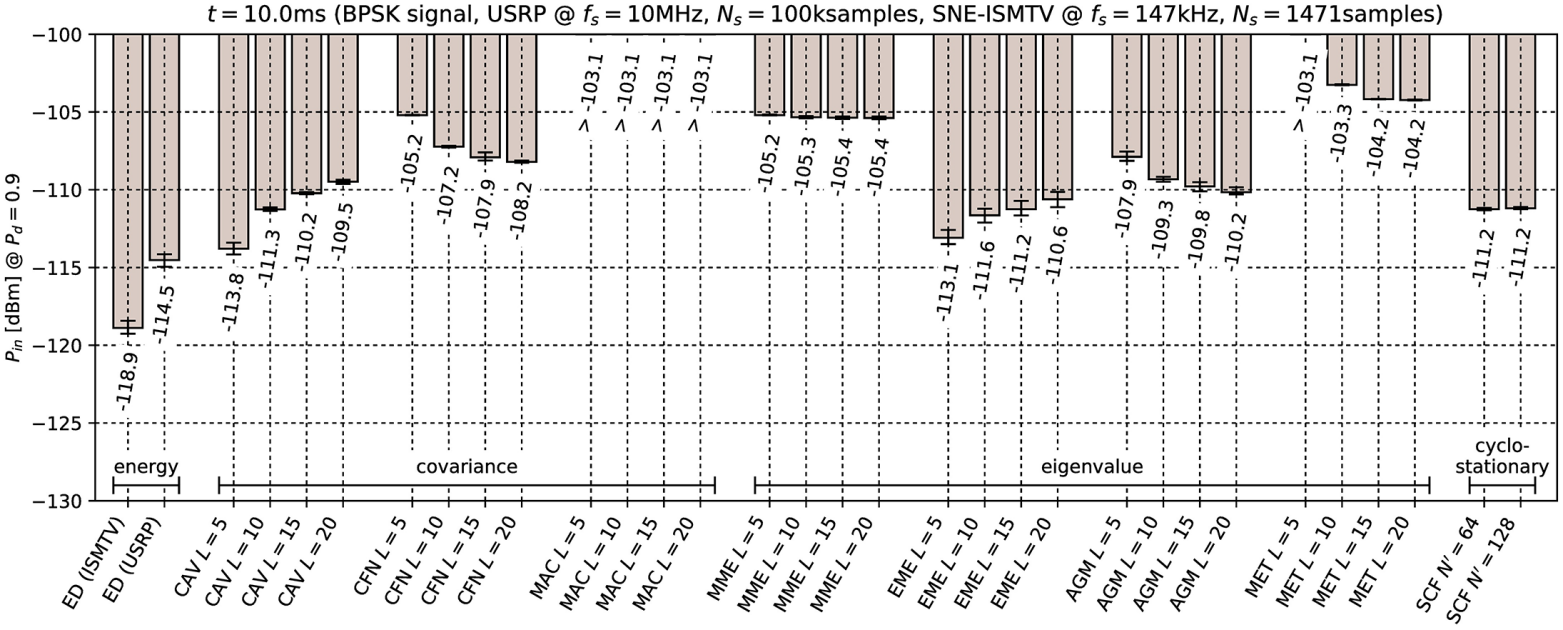
Lowest detectable power *P*_*in*_ of the BPSK signal for various methods with sensing time *t* = 10.0ms. Error bars show binomial confidence intervals for *α* = 10^−6^.

Fig 6 through 11 show the lowest detectable power *P*_*in*_ for each sensing method. Figs 6–8 show detection of the microphone signal when using the settings A, B and C respectively. Figs 9–11 show detection of the BPSK signal when using the settings A, B and C respectively.

The results of the experiment show that energy detector was able to detect weaker signals than other methods under all tested conditions. This is in contrast to simulations in [10–12], where the energy detector with known noise floor had worse performance than covariance detection. Similarly, simulations in [13] showed that a cyclostationary detector had significantly better performance than energy detection with a static threshold. This suggests that the noise in our setup did not consist solely of uncorrelated white thermal noise, which is the noise model used in these simulations. Noise sources, like the undesired coupling of signals in the circuit, could account for correlation and periodicity in noise samples.

Device settings have a significant effect on some methods. For instance, the MAC method is among the best performing methods for detecting the microphone signal with setting A and worst with setting C. On the other hand, EME and the SCF method for *N*′ = 128 produced consistently good results across all settings. The results for the CAV method with setting C also show that increasing the smoothing factor *L* does not always lead to better performance.

Our results show that most methods perform worse when detecting the BPSK signal compared to the microphone signal. The most affected were the covariance- and eigenvalue-based methods with lower sampling rates (settings A and B). With setting A, CAV, CFN and MET methods were not able to detect the signal with the chosen probability *P*_*d*_ even with the highest signal generator output power used in the experiment. We can attribute at least part of this effect to the fact that the BPSK signal occupies a significantly wider bandwidth than the microphone signal. This is consistent with theoretical analysis in [11]: Covariance- and eigenvalue-based methods rely on sample correlations for detection. These correlations appear when the bandwidth of the detected signal is significantly narrower than that of the receiver.

Cyclostationary detection performed well in comparison to other methods even with the lowest sampling rate, which is in contrast with findings in [16]. The SCF method also exhibited lower sensitivity with the BPSK signal than with the microphone signal, although the effect was less significant than with the covariance-based methods.

As expected, the results for energy detection methods were almost identical for both test signals. The performance of energy detection with USRP varies slightly between different settings. This can be attributed to the fact that total thermal noise power varies with the channel bandwidth. Therefore the energy detector’s signal-to-noise ratio, and with it its minimal detectable power, is inversely proportional to the sampling frequency. Similarly, minimal detectable power is also inversely proportional to the number of samples. These two factors match the seen variation to within 0.5 dB.

From the results it can also be seen that the analog implementation of energy detection consistently outperformed the digital implementation. It should be noted that SNE-ISMTV-UHF with a fixed channel bandwidth of 1.7 MHz had an advantage over USRP for settings B and C, which had broader channels and therefore also higher thermal noise power. The simpler SNE-ISMTV-UHF receiver also likely has a lower noise figure than the USRP.

### 9.2 Comparing noise level sensitivity

The results of this experiment are shown in Table 4. The energy detector only tolerated minimal noise power changes without a significant increase in *P*_*fa*_, which is consistent with theory. The EME method, which also uses signal energy in its test statistic, suffered from a similar problem as the energy detector. All other methods under evaluation (SCF, CAV, CFN, MAC, MME, AGM and MET) tolerated a larger than 40 dB increase in noise power without a significant effect on *P*_*fa*_.

**Table 4:** Sensitivity to changes in noise floor for various methods.

*f*_*s*_	1MHz	2MHz	10MHz
*N*_*s*_	25ksamples	25ksamples	100ksamples
Method	Δ*P*_*noise*_[dB] @ *P*_*fa*_ = 0.2		
ED (USRP)	**0.2**	**0.2**	**0.2**
EME *L* = 5	**35.9**	**0.7**	**1.2**
EME *L* = 10	**0.7**	**0.3**	**0.5**
EME *L* = 15	**0.6**	**0.2**	**0.4**
EME *L* = 20	**0.6**	**0.2**	**0.4**
others	>40.0	>40.0	>40.0

### 9.3 Effect of noise compensation

**Fig 12:**
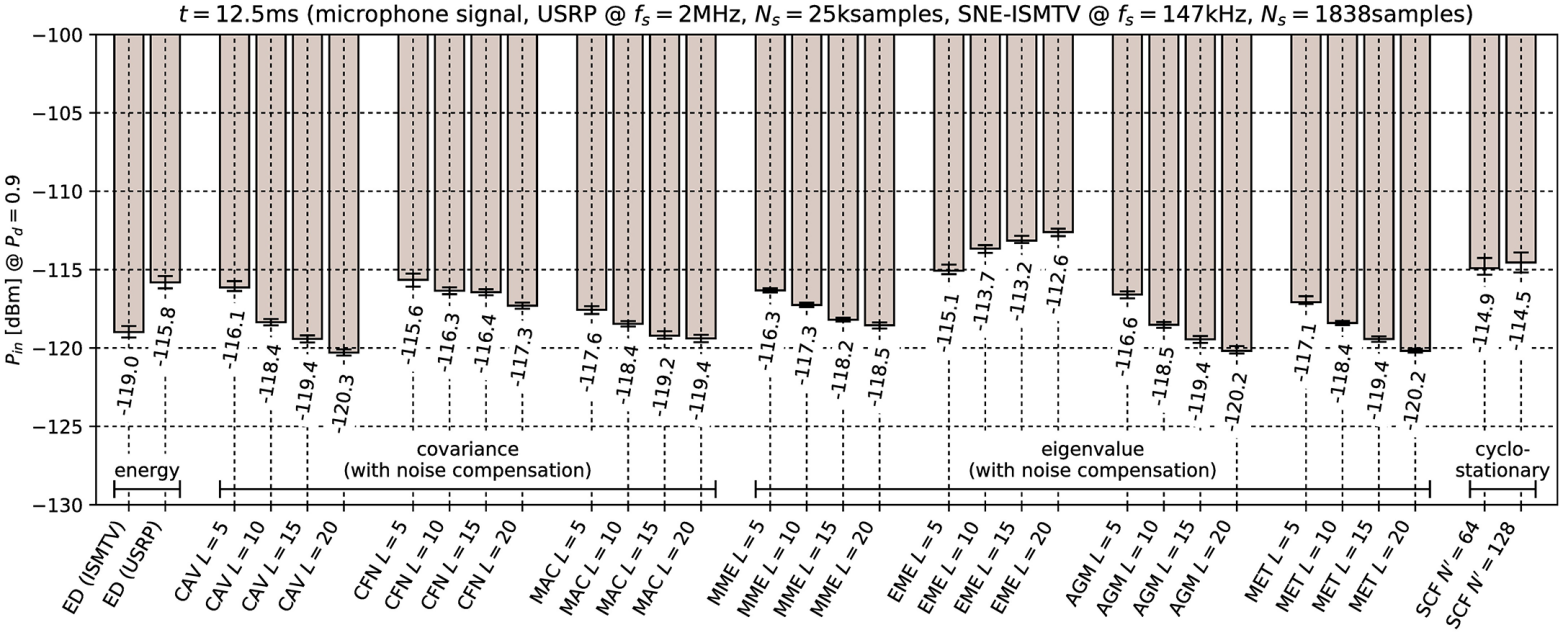
Lowest detectable power *P*_*in*_ of the microphone signal when noise compensation is used with covariance-based methods. Error bars show binomial confidence intervals for *α* = 10^−6^.

**Fig 13:**
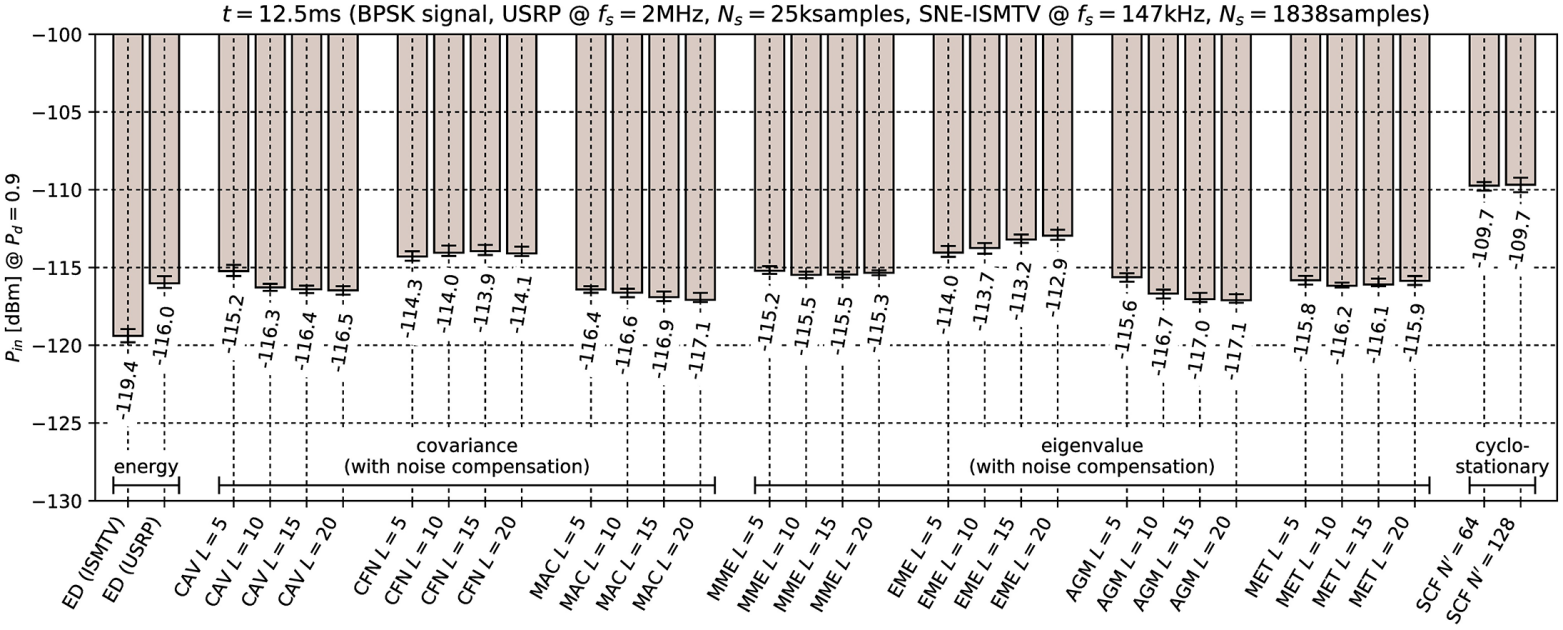
Lowest detectable power *P*_*in*_ of the BPSK signal when noise compensation is used with covariance-based methods. Error bars show binomial confidence intervals for *α* = 10^−6^.

The results of the minimal detectable signal test with noise compensation enabled are shown in Figs 12 and 13 for the microphone and BPSK signals respectively. It can be seen that noise compensation decreases the minimal detectable signal power for covariance- and eigenvalue-based methods by 7.3 to 14.1 dB, depending on the test statistic. The only exception was the EME method, which was not significantly effected by the noise compensation. *This result supports our earlier conclusion that the relatively low performance of covariance methods without noise compensation is due to correlations in the USRP receiver noise samples.*

**Fig 14:**
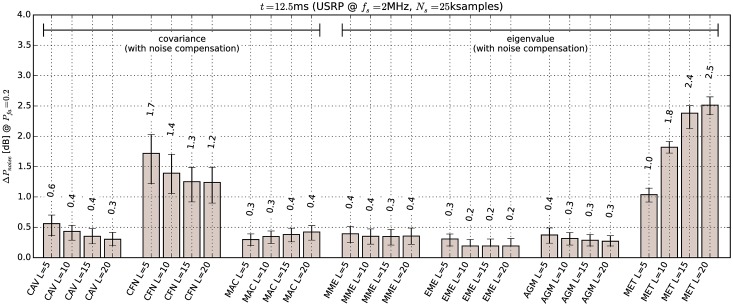
Sensitivity to changes in noise floor for covariance-based methods with noise compensation. Error bars show binomial confidence intervals for *α* = 10^−6^.

The results of the noise level sensitivity test are shown in Fig 14. Comparison of these results with those in Table 4 indicates that with noise compensation most covariance methods become extremely sensitive to noise level. The most insensitive was the MET detector which tolerated only 2.5 dB increase in noise level. This is consistent with [33] which predicts that static pre-whitening filters will not be effective.

This outcome can be explained by the fact that noise compensation matrix **Q** was pre-calculated from samples with signal generator turned off and hence only took into account the colored noise generated internally by the USRP receiver. External noise applied in this experiment was white Gaussian noise. When added to internal USRP noise, Gaussian noise produced different correlations in signal samples than those compensated for. Hence from the standpoint of the compensated covariance detectors, adding external Gaussian noise had the same effect as adding signal with correlated samples.

### 9.4 Comparing computational complexity

**Fig 15:**
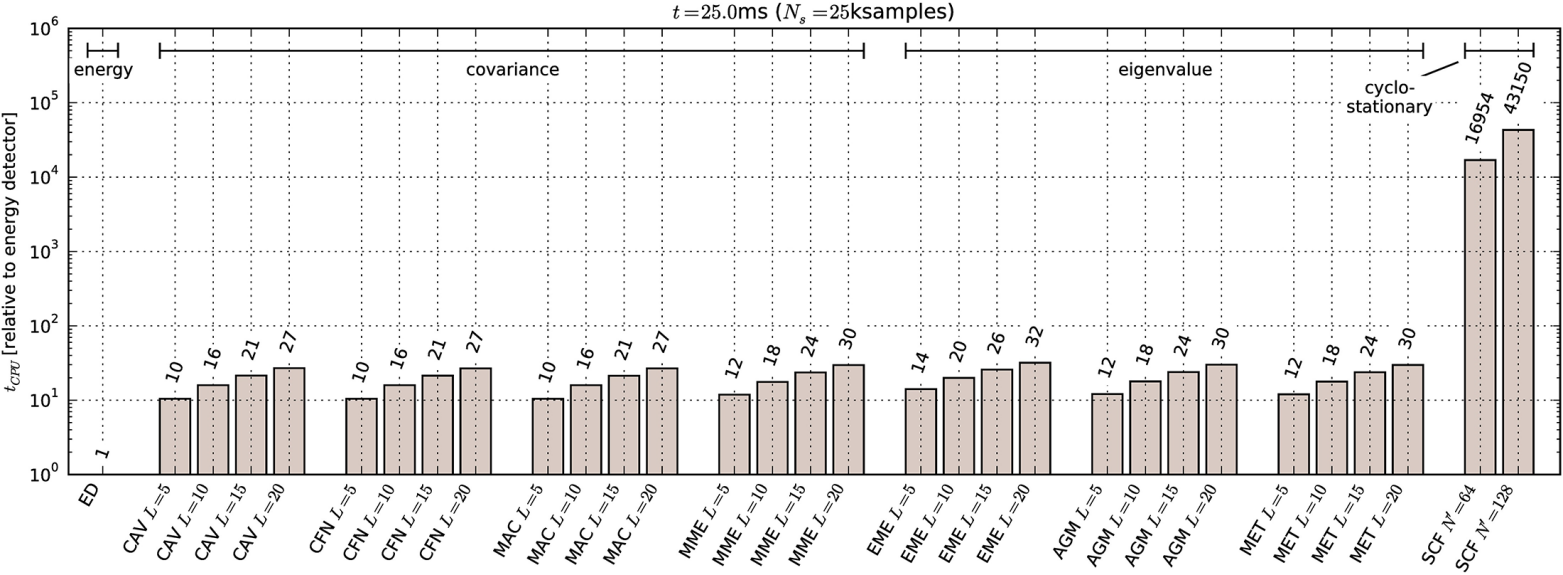
Processing time required for calculation of test statistic *γ*.

**Fig 16:**
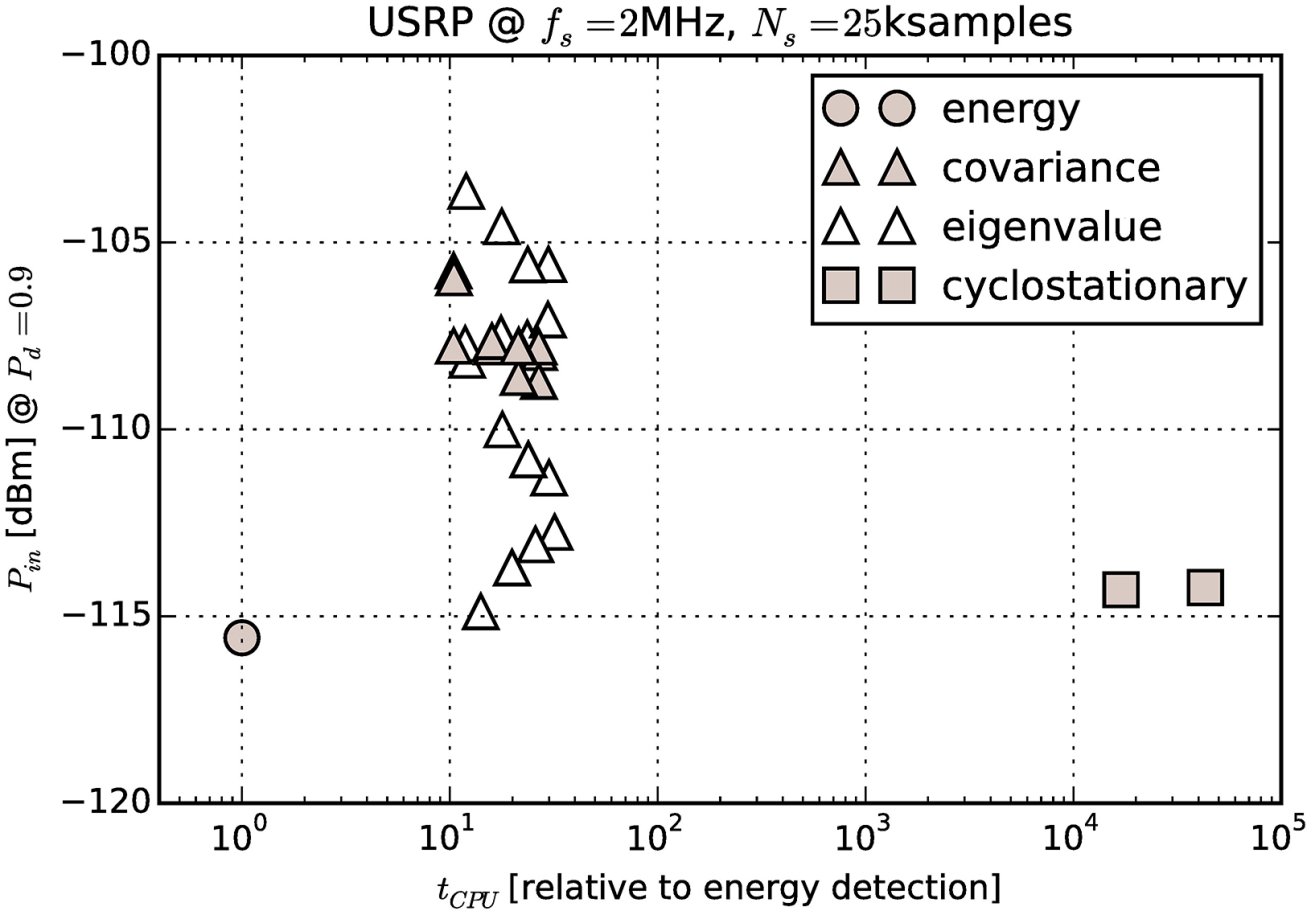
Comparison of processing time versus minimal detectable power.

**Fig 17:**
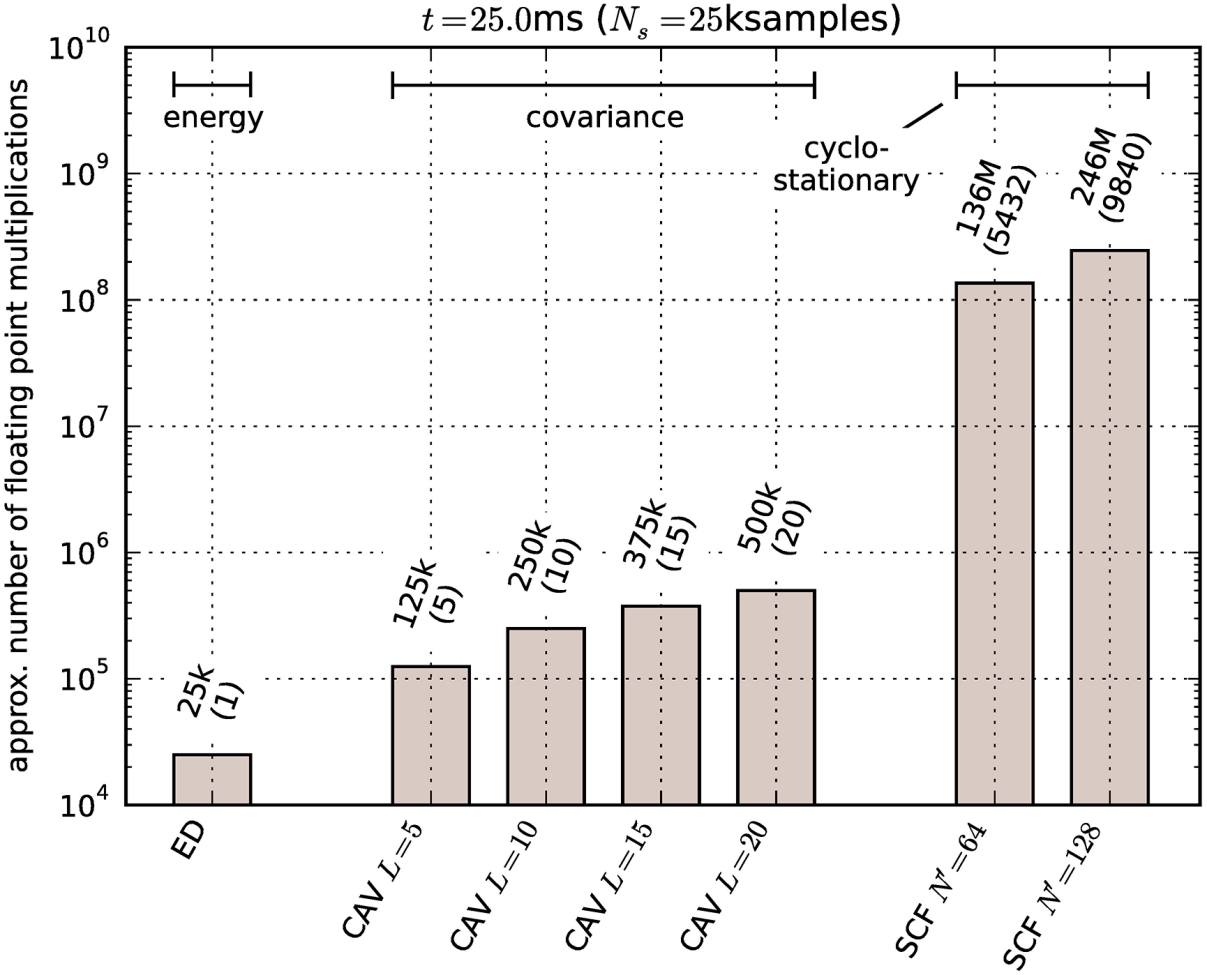
Floating point multiplications required for calculation of test statistic *γ*. Numbers in parenthesis are relative to energy detection. Approximations for other covariance-based detectors are equal to CAV.

Benchmark results are shown in Fig 15. Time required to calculate *γ* is compared to minimal detectable power results from Section 9.1 in Fig 16. For comparison, we have also approximated the number of required floating point multiplications for each detector, which is a common way of specifying algorithm complexity. The equations used for approximations are listed in Table 5. Floating point multiplication counts for specific settings used in our experiment are compared graphically in Fig 17.

**Table 5:** Approx. number of floating point multiplications.

Energy	*N*_*s*_	Squaring operations in (6)
Covariance-based	*LN*_*s*_	Covariance estimation in (7) [34]
Cyclostationary	2*N*′^2^ *P* log_2_ *P*	Second set of complex FFTs [36]

From the figures it can be seen that measurements do in fact follow the general trends predicted by theory. For example, the value of *L* significantly affects the processing time required for covariance-based detectors. There is also very little difference in the time required for test statistics that involve calculation of eigenvalues, confirming the assumption that eigenvalue calculation time is negligible. We can also see that cyclostationary detection is many orders of magnitude slower than energy detection or covariance-based detection. This is in contrast with results in [10] where covariance-based methods were found to have very high complexity.

However, ratios between energy detection and other methods of detection do not fit well. That can be explained by the fact that processing time of a script in a high-level programming language like Python is affected by many complicated factors, not only the number of multiplications. For example, choosing different vectorization methods in NumPy can significantly affect the processing time due to interpreter overhead and processor cache effects.

The Python interpreter has a significant overhead compared to native code. Even though the NumPy module contains optimized code for matrix operations, we believe these results are not an absolute measure of how optimized native code implementations would perform. Hence they are only useful for a relative comparison, i.e. to compare the methods against each other. Therefore we have normalized the results to the execution time of the energy detector.

## 10 Conclusions

We proposed a unified methodology for objective quantitative evaluation of signal detection methods suitable for simulation or experimental set-ups. We elaborated on the methodology and performed the most comprehensive *experimental evaluation* to date. We evaluated nine selected detection methods with a digital and one of them also with an analog implementation. We implemented the energy detection, covariance- and eigenvalue-based methods and cyclostationary detection using a USRP software-defined radio device. We also evaluated a custom implementation of an analog energy detector. We compared minimal detectable power of a wireless microphone signal and a BPSK signal for a fixed probability of detection, effect of changes in noise power on the probability of false alarm and the computational complexity of signal detection methods.

The results of our evaluation contradict some of the previously published works comparing signal detection methods based on individual evaluations. We show that cyclostationary detectors can perform well even with low sampling rates and number of samples and that covariance- and eigenvalue-based detection do not necessarily have a high computational complexity compared to other methods. We conclude that this validates our premise that a unified methodology is valuable in obtaining reliable and reproducible comparisons of signal detection methods.

Furthermore, our results show that experimental results for covariance-, eigenvalue-based and cyclostationary methods can differ significantly from simulations due to noise coloring effects of real receivers. In our results, under no noise uncertainty, energy detection outperforms other methods in terms of minimal detectable power. While these effects are known and have already been theoretically analyzed in existing literature, many published simulations do not take them into account. This again demonstrates the importance of testing signal detection methods in realistic environments.

Based on the discussion in Section 2, we believe that the theoretical and practical contributions of this paper are relevant to a broad range of researchers. The generic methodology can be used in textbooks and educational materials and can also be applied in university and industrial labs for objective and meaningful evaluations. The experimental findings complement existing surveys and shed new insights on existing methods.
